# Investigation of the Sensitivity and Specificity of Laboratory Tests Used in Differential Diagnosis of Childhood Brucellosis

**DOI:** 10.7759/cureus.6756

**Published:** 2020-01-23

**Authors:** Halil Kazanasmaz, Süleyman Geter

**Affiliations:** 1 Pediatrics, Harran University Medical School, Sanliurfa, TUR; 2 Pediatrics, Sanliurfa Training and Research Hospital, Sanliurfa, TUR

**Keywords:** brucellosis, c reactive protein, differential diagnoses, erythrocyte sedimentation rate

## Abstract

Objectives

Childhood brucellosis is a common public health problem in developing countries. The diagnosis of brucellosis based on nonspecific symptoms is an ongoing problem for physicians, especially in endemic areas. In this study, it is aimed to discuss the efficacy of frequently used test methods in the differential diagnosis of brucellosis.

Methods

The records of 332 patients admitted to pediatric clinic on suspicion of brucellosis were retrospectively analyzed. Patients with brucellosis were included in the positive group (n = 262) and those without brucellosis were included in the negative group (n = 70).

Results

As a result of biochemical analysis of the cases, median alanine aminotransferase (ALT), aspartate aminotransferase (AST), gamma-glutamyl transferase (GGT), erythrocyte sedimentation rate (ESR), and C reactive protein (CRP) values were significantly higher in the positive group than that in the negative group (p<0.05). There was no significant difference between median white blood cell, neutrophil, lymphocyte, neutrophil to lymphocyte ratio, hemoglobin, and platelet values between groups (p>0.05). Receiver operating curves were plotted to compare predictive values of CRP (area under curve (AUC): 0.635, p= 0.001), ESR(AUC:0.701, p<0.001), AST(AUC: 0.595, p=0.015), ALT(AUC:0.604, p=0.007), and GGT(AUC:0.593, p=0.016) in 332 patients with suspected brucellosis.

Conclusions

Increased levels of AST, ALT, GGT, CRP, and ESR may have a complementary role in the differential diagnosis of childhood brucellosis. However, all of these markers should be evaluated with clinical findings due to their low specificity and sensitivity.

## Introduction

Childhood brucellosis is a zoonotic disease transmitted to humans through infected animals [1]. Brucellosis confronts public health currently as a widespread problem all over the world and it is especially common in the Middle East including Turkey [1-2]. The incidence of this zoonotic disease in Turkey is around 23-25 per 100,000 annually [1,3]. Brucellosis is frequent in the rural areas of Turkey, especially in Central and Southeastern Anatolia, where animal husbandry is common. Among the causative strains, *Brucella melitensis* is the most commonly isolated strain [1,3].

Animals and animal products are the main sources to cause brucellosis in humans. Contamination occurs via the consumption of unpasteurized dairy products and/or raw meat and through direct contact with infected animals in the presence of erosions on the skin and mucosa. Farmers, veterinarians, abattoir workers, and workers in animal husbandry or people in contact with animals are at risk of infection. If those animals are kept close to the living environment of humans, animal exposure will potentially be inevitable and the families of these people will be at high risk, too [4]. Fever and monoarthritis, often involving the hip and/or knee joint are common clinical findings in brucellosis; however, no disease-specific pathognomonic signs and symptoms exist [5-7]. Although rarely, brucellosis can be associated with a variety of clinical findings; including acute renal failure, endocarditis, splenic abscess, spondylitis, and encephalitis [4,8-9]. Laboratory tests can often yield normal inflammatory biomarker levels [5].

The definitive diagnosis of brucellosis is made by isolating the microorganism from the blood or various tissue samples (such as the bone marrow, synovial fluid, etc.) or by identifying the causative agent based on the results of molecular tests like polymerase chain reaction (PCR) [10]. Besides the most commonly used diagnostic clinical laboratory test methods including Rose Bengal plate agglutination (RBPT), standard tube agglutination test (SAT), coombs antiglobulin test, and enzyme-linked immunosorbent assay; blood culture is recognized as the gold standard test [11-12]. Diagnosing brucellosis is challenging because the diagnostic methods have advantages and disadvantages over each other; no clinical pathognomonic findings have been described yet, and the levels of inflammatory biomarkers can be normal [5,12-13].

This study aimed to discuss the efficacy of commonly used test methods in the diagnosis of brucellosis in light of the information in the literature and to demonstrate the clinical characteristics in pediatric patients from Şanlıurfa province, where brucellosis is endemic.

## Materials and methods

Patients, who were admitted to the pediatric clinics of two hospitals in Şanlıurfa province due to clinical suspicion of brucellosis in the period from January 2018 to December 2018, were included in the study. Before the study, written informed consent was obtained from the parents of the patients who participated in this study. This study conformed to the principles of the 2008 Declaration of Helsinki and was approved by the local ethics committee in Turkey. Immunological diagnosis of brucellosis was made using serological methods. All patients underwent RBPT and SAT analyses simultaneously. RBPT positive patients were included in the study. Patients were accepted positive for brucellosis when both titers were 1/160 or more in two SAT tests performed consecutively at two-week intervals besides a positive RBPT result. Patients were excluded when the first titer was positive but the second titer was negative. Patients were accepted negative despite RBPT positivity when SAT negative patients (<1/160) had negative SAT results (1/160) again in the following second week; additionally to negative blood culture results and no clinical suspicion of brucellosis. 

Collection of Blood Samples

Blood culture samples were collected from peripheral antecubital veins appropriately. A total of 2 ml of the blood sample was placed in one aerobic and one anaerobic blood culture tubes under sterile conditions. The BD BACTEC ™ FX40 blood culture system (Becton Dickinson Diagnostics, Sparks, MD, USA) was used for isolating microorganisms from the blood. A blood sample volume of 2 mL was placed in gel-containing tubes for biochemical analyses and tube agglutination test, and into K2 EDTA tubes (potassium-2 ethylene diamine tetraacetic acid) for laboratory tests, absolute blood count, and erythrocyte sedimentation rate (ESR) measurements according to the manufacturer's instructions. ESR measurements were obtained with an automatic Vision (Shenzhen YHLO Biotech, China) device. Complete blood counts were calculated with an automatic blood cell counting device (Abbot Celldyn 3500 Ill, USA). As a result of the analysis, white blood cell (WBC), neutrophil (NEU), lymphocyte (LYM), hemoglobin (Hb), and platelet (PLT) values were obtained. Neutrophil to lymphocyte ratio (NLR) was obtained by dividing the absolute NEU count by the absolute LYM count. Biochemical analysis results were obtained using a spectrophotometric chemical analyser Architect C16000 (Abbott Diagnostics, Abbott Park, USA). As a result of the analysis, alanine aminotransferase (ALT), aspartate aminotransferase (AST), C reactive protein (CRP), and gamma-glutamyl transferase (GGT) values were obtained.

Rose Bengal Plate Agglutination Test

The standard method for RBPT was performed on a glass plate [14]. Blood serum samples of 30 ul antigen and 30 ul serum were placed onto a glass slide and mixed well at room temperature. Any degree of agglutination visible to a naked eye within four minutes was considered positive.

Standard Tube Agglutination Test

The serum in the blood samples was tested in glass tubes at the following dilutions of 1/40, 1/80, 1/160, 1/320, 1/640, 1/1280, and 1/2560 using the Cromatest (Linear Chemicals Montgat, Spain) kit according to the manufacturer's instructions. Patients with a titer of 1/160 and above were considered positive.

Analysis of the Data

Statistical analyses were performed using the SPSS 24.0 version (SPSS Inc., Chicago, IL) package program. Descriptive statistics were summarized as median, minimum, and maximum values. The suitability of the variables to the normal distribution was investigated using visual (histogram and probability charts) and analytical methods (Kolmogorov-Smirnov, Shapiro-Wilk tests). Mann-Whitney U test was used to analyze the data of the groups that were not homogeneous and had a different number of cases. Categorical variables were analyzed using Pearson chi-square test. Specificity and sensitivity analyses were performed using receiver operating curve (ROC) analysis method. In the ROC analysis, the area under curve (AUC) values were studied. The comparisons were considered to be statistically significant when the probability (p) value in the study analysis was less than 0.05.

## Results

This study included 332 patients, who were referred to pediatric clinics with suspicion of brucellosis. While 78.9% of the study participants were positive, 21.1% were negative (Table 1). In the positive group, 52.3% were males and 47.7% were females while in the negative group, 54.3% were males and 47.3% were females. The comparison of the two groups with the Pearson chi-square test revealed no significant differences in gender distribution (p = 0.766). The mean age of the patients in the positive group was 10.48 ± 4.48 (min-max, 1-18) years, whereas the mean age of the patients in the negative group was 9.69 ± 4.39 (min-max, 1-18) years. The Mann-Whitney U test did not reveal any differences in the age distribution between the two groups (p=0.165).

**Table 1:** Sociodemographic and clinical characteristics of cases ^a^:Mann–Whitney U test;^ b^: Pearson chi-square test

	Positive group (n=262)	Negative group (n=70)	P value
Age/Year median(min-max)	11(1-18)	9(1-18)	0.165^a^
Gender M/F n(%)	137(52.3)/125(47.7)	38(54.3)/32(45.7)	0.766^b^
Seasons n(%)			
Spring	34(13)	16(22.9)	0.006^b^
Summer	94(35.9)	13(18.6)	
Autumn	88(33.6)	21(30)	
Winter	46(17.6)	20(28.6)	
Rural and Farming / Urban n(%)	216(82.4)/46(17.6)	41(58.6)/29(41.4)	<0.001^b^
Case in family Positive/Negative n(%)	64(24.4)/198(75.6)	5(7.1)/65(92.9)	0.002^b^
Unpasteurized milk consumption Positive/Negative n(%)	165(63)/97(37)	27(38.6)/43(61.4)	<0.001^b^

Patients with positive test results (35.9%) were found out to present to the hospital mostly in summer, while autumn was the most common admission season (30%) in the negative group. A significant difference in the seasonal distribution of the cases was found between the two groups based on the Pearson chi-square test results (p = 0.006). While 24.4% of the positive group had brucellosis in the family history, 7.1% of the negative group had brucellosis in their family history. A significant difference was found in the frequency of family history of brucellosis between the groups (p = 0.002) (Table 1). Of the study patients, 63% of the positive group and 38.6% of the negative group reported consumption of unpasteurized milk and dairy products in medical history. The frequency of consumption of unpasteurized milk and dairy products were significantly different between the groups (p <0.001). While 82.4% of the cases in the positive group lived in rural areas and engaged in animal husbandry, this rate was 58.6% in the negative group. The frequency of study participants living in rural areas and working in animal husbandry was significantly different between the two groups (p <0.001).

In the positive group with the brucellosis diagnosis; arthralgia (45.8%), fever (≥38.5 C^0^) (28.6%), and malaise and fatigue (11.5%) were the most common complaints at the time of presentation to the hospital (Table 2). In the positive group, the physical examination findings were nonspecific in 50.4% of the cases, while 32.4% of the patients had arthritis and 11.5% had hepatosplenomegaly. Of the patients in the positive group, 13.4% were hospitalized and 86.6% of them were treated as outpatients. Brucellosis was isolated in the blood cultures in only 4.2% of the positive group.

**Table 2:** Clinical features of positive cases with brucellosis STA: Standard tube agglutination test.

(n=262)	Number of cases, n /%
Complaints n(%)	
Arthralgia	120(45.8)
Fever (≥38.5 C^0^)	75 (28.6)
Malaise and fatigue	30(11.5)
Abdominal pain	19(7.3)
Night sweating	9(3.4)
Weight loss	9(3.4)
Physical examination n(%)	
Arthritis	85(32.4)
Hepatomegaly	17(6.5)
Splenomegaly	13(5)
Lymphadenopathy	11(4.2)
Nonspecific rash	4(1.5)
No findings	132(50.4)
Treatment n(%) Hospitalization / Outpatient	35(13.4)/227(86.6)
STA titer n(%)	
1:160	70(26.7)
1:320	57(21.8)
1:640	114(43.5)
1:1280	17(6.5)
1:2560	4(1.5)

Laboratory analysis results of the positive and negative groups were compared with the Mann-Whitney U test. There were no significant differences between the groups in the median WBC, LYM, NEU, NLR, Hb, and PLT counts revealed by the hematological analysis (p> 0.05) (Table 3). The analysis of the biochemical test results revealed that the median AST, ALT, GTT, ESR, and CRP values were significantly higher in the positive group compared to the negative group (p< 0.05) (Table 3).

**Table 3:** Comparison of laboratory analysis of positive and negative groups ^a^: Mann–Whitney U test; WBC: White blood cell; NEU: Neutrophil; LYM: Lymphocyte; Hb:Hemoglobin; PLT: Platelets; GGT: Gamma-glutamyl transferase; ALT: Alanine aminotransferase; AST: Aspartate aminotransferase; CRP: C reactive protein; ESR: Erythrocyte sedimentation rate; NLR: Neutrophil to lymphocyte ratio

	Positive group (n=262)	Negative group (n=70)	P value^a^
WBC (10e3/µL)	8.3(1.85-70.37)	8.5(4.91-19.2)	0.603
NEU (10e3/uL)	3.6(0.6-14.78)	4(1.10-12.98)	0.129
LYM (10e3/uL)	4(0.97-11.9)	4(1.7-11.9)	0.860
NLR	0.99(0.22-6.35)	1.08(0.15-4.45)	0.353
Hb (g/dL)	13(9.5-16.4)	13.4(10.5-15)	0.406
PLT (10e3/µL)	303.65(13.81-651)	325(171-452.36)	0.469
ALT (U/L)	24.5(8-547)	21(6-85)	0.007
AST (U/L)	32(7-569)	28(12-69)	0.015
GGT (U/L)	14(8-128)	11(9-51)	0.016
CRP (mg/dL)	0.3(0-15.94)	0.18(0-7.14)	<0.001
ESR (mm/h)	21(5-116)	13(5-88)	<0.001

**Figure 1:**
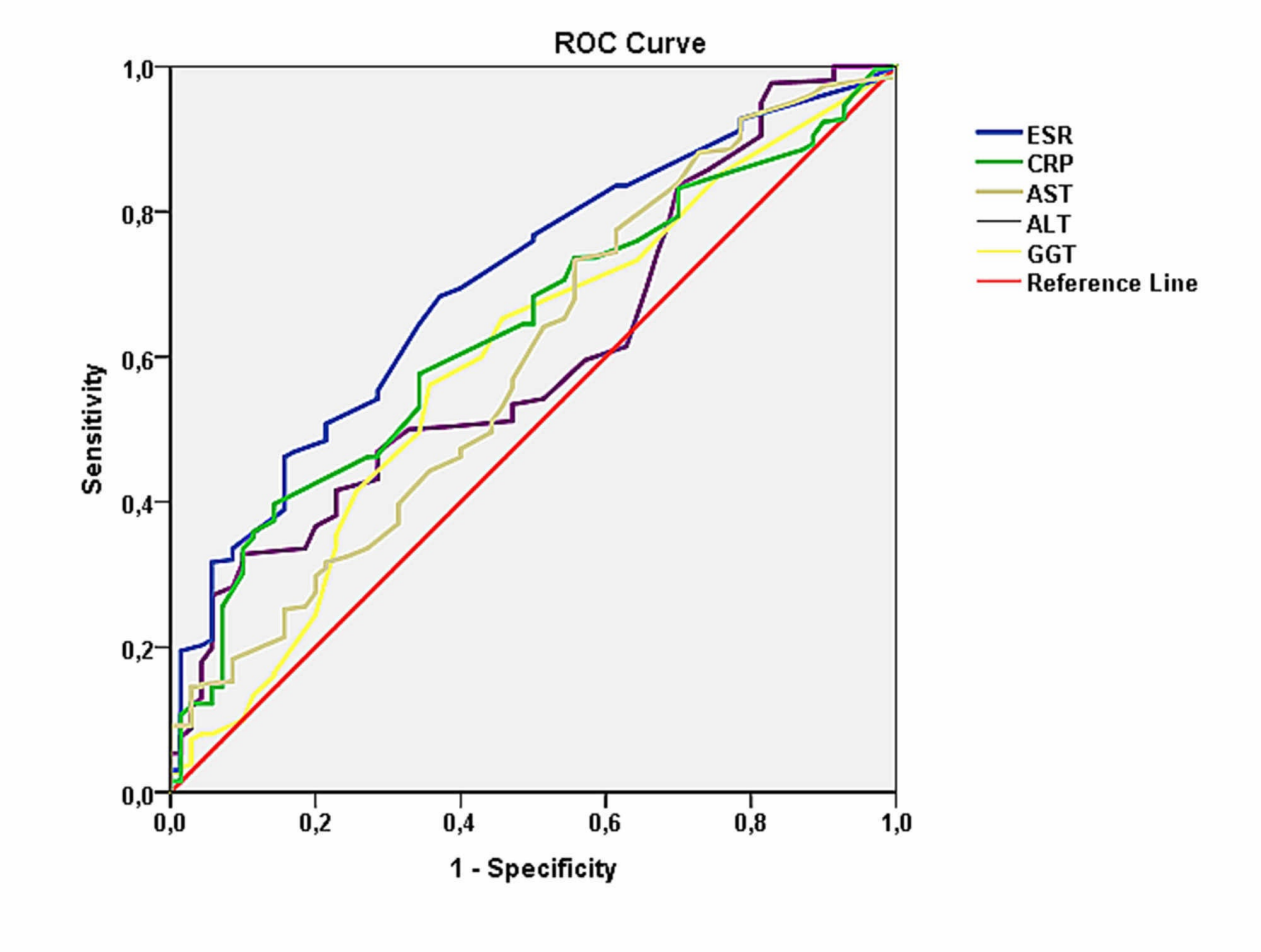
Comparison of specificity and sensitivity with receiver operating characteristic graph ROC: Receiver Operating Curve; GGT: gamma-glutamyl transferase; ALT: alanine aminotransferase; AST: aspartate aminotransferase; CRP: C reactive protein; ESR: erythrocyte sedimentation rate

ROC curves were drawn to compare the predictive values of the laboratory test results of the 332 subjects included in the study (Figure 1). The analysis revealed that AUC for AST was 0.595 and the p-value was 0.015, AUC for ALT was 0.604 and the p-value was 0.007, AUC for GGT was 0.593 and the p-value was 0.016, AUC for CRP was 0.635 and the p-value was 0.001, and AUC for ESR was 0.701 and the p-value was < 0.001 (Table 4).

**Table 4:** ROC analysis results for AST, ALT, GGT, ESR, and CRP measurements ROC: Receiver Operating Curve; AUC: Area under the curve; GGT: Gamma-glutamyl transferase; ALT: Alanine aminotransferase; AST: Aspartate aminotransferase; CRP: C reactive protein; ESR: Erythrocyte sedimentation rate; WBC: White blood cells; NEU: neutrophil; LYM: lymphocyte; NLR: Neutrophil to lymphocyte ratio; PLT: platelet.

	Values	AUC	Sensitivity	Specificity	P value
ALT (U/L)	21.5	0.604	53.4	52.9	0.007
AST (U/L)	31.5	0.595	53.1	54.3	0.015
GGT (U/L)	12.5	0.593	59.9	57.1	0.016
CRP (mg/dL)	0.21	0.635	57.6	65.7	0.001
ESR (mm/h)	15.5	0.701	68.3	62.9	<0.001
WBC (10e3/µL)	-	0.480	-	-	0.604
NEU (10e3/µL)	-	0.441	-	-	0.130
LYM (10e3/µL)	-	0.507	-	-	0.860
NLR	-	0.464	-	-	0.353
PLT (10e3/µL)	-	0.472	-	-	0.469

## Discussion

Brucellosis is a systemic infectious disease that can be manifested by various clinical pictures [15-20]. Although brucellosis is well-controlled in developed countries, the disease remains to be a major health problem in developing countries including Iran and Turkey [16,20]. Brucellosis is especially seen in the Eastern and Southeastern Anatolia regions of Turkey, where livestock farming is common [15]. The disease is more common in boys at the age range from 9 to 11 years born to families engaged in animal husbandry in rural areas, where consumption of unpasteurized milk and dairy products is widespread [16,19]. In our study, 52.3% of the patients positive for brucellosis were males at a mean age of 10.48 ± 4.48 years. Of the brucellosis patients, 63% consumed unpasteurized milk and dairy products and 82.4% of them lived in the countryside with their families engaged in animal husbandry. Diagnosing brucellosis based on nonspecific disease symptoms is still an issue for physicians, especially working in endemic areas. While fever, night sweats, joint pain, malaise, and fatigue are nonspecific symptoms of brucellosis; arthralgia, hepatomegaly, and myalgia are the most common complaints [11,16,20]. Similarly, in our study, the main complaints were arthralgia, fever, malaise, and fatigue.

Clinical manifestations of brucellosis may range from nonspecific symptoms such as long-term fever, fatigue, and malaise to local organ involvement such as arthritis and neurobrucellosis [21]. Therefore, many studies have been carried out to determine the laboratory parameters, which will support clinical findings. Anemia, leukopenia, and especially in endemic areas thrombocytopenia can be detected in the complete blood count in patients with brucellosis [19-22]. Additionally, it has been reported that increased levels of inflammatory markers such as CRP, ESR, and liver enzymes such as AST, ALT, and GTT in biochemical tests can support the diagnosis [23-27]. A study by Bozdemir et al. reported that increased NLR values could be used as an indicator of inflammation in childhood brucellosis [22]. Another study by Aktar et al. reported that NLR may reach abnormally high levels in children with brucella arthritis [5]. In our study, there were no significant differences in the median WBC, NEU, LYM, NLR, and PLT values between the brucellosis positive and negative groups, whereas the median AST, ALT, GGT, CRP, and ESR values were significantly higher in the positive group. Similarly, none of the WBC, NEU, LYM, NLR, and PLT values were found to be significant in the ROC analysis (p >0.05) (Table 4). These results suggested that WBC, NEU, LYM, NLR, and PLT values might not always be used as supportive parameters in the differential diagnosis of childhood brucellosis. Although the ROC analysis yielded significant results for the AST, ALT, GGT, CRP, and ESR levels; the specificity and sensitivity of these parameters did not reach adequate levels (p <0.05) (Table 4). The ROC analysis revealed significant results for an ESR cut-off value of 15.5 mm/g with 68.3% sensitivity and 62.9% specificity. These results suggest that high levels of AST, ALT, GGT, CRP, and ESR can be used as adjunct parameters in the differential diagnosis of childhood brucellosis. However, the role of these markers in the differential diagnosis is limited due to their low specificity and sensitivity.

Limitation of the Study

The major limitation of this study is that the control group did not consist of healthy children. We thought that this limitation might have contributed to the absence of a significant difference in the complete blood count parameters between the brucellosis positive and negative groups. Also, we think that the low number of blood culture-positive cases might have contributed to the low specificity and sensitivity found for the inflammation markers in ROC analysis.

## Conclusions

In developing countries where consumption of unpasteurized milk and dairy products is widespread, brucellosis is common especially in boys born to families engaged in animal husbandry in rural areas. A potential diagnosis of brucellosis should be remembered in the presence of joint involvement, prolonged fever, and organomegaly. Despite the insufficient levels of specificity and sensitivity, we are of the opinion that high levels of AST, ALT, GGT, CRP, and ESR may have a complementary role in the differential diagnosis of childhood brucellosis. 
